# 厄洛替尼治疗脑脊液检测*EGFR*敏感突变的肺腺癌一例

**DOI:** 10.3779/j.issn.1009-3419.2016.01.07

**Published:** 2016-01-20

**Authors:** 晓燕 李, 辉 杨, 华艳 许, 沙沙 王, 红军 高

**Affiliations:** 100071 北京，中国人民解放军第307医院肺部肿瘤科 Department of Lung Cancer, 307 Hospital of People's Liberation Army, Afliated to Academy of Military Medical Sciences, Beijing 100071, China

**Keywords:** 肺肿瘤, 脑转移, 脑脊液, 表皮生长因子受体酪氨酸激酶抑制剂, 客观有效率, Lung neoplasms, Brain metastasis, Cerebrospinal fluid, Epidermal growth factor receptor tyrosine kinase inhibitors, Objective response rate

## Abstract

**背景与目的:**

脑是非小细胞肺癌(non-small cell lung cancer, NSCLC)常见的转移部位。有研究显示表皮生长因子受体酪氨酸激酶抑制剂(epidermal growth factor receptor tyrosine kinase inhibitors, EGFR-TKI)可透过血脑屏障，发挥抗肿瘤作用。本例报道采用突变扩增阻滞系统(amplifcation refractory mutation system, ARMS)检测脑脊液EGFR突变指导临床治疗的可行性，并分析TKI治疗肺癌脑转移的疗效和安全性。

**方法:**

腰穿取得脑脊液标本，检测颅内压力，检验常规、生化及肿瘤标志物，查找脱落细胞，采用ARMS法检测EGFR基因突变，得到阳性结果后给予盐酸厄洛替尼(erlotinib，tarceva，特罗凯)治疗，150 mg，每天1次。按照实体瘤疗效评价标准1.1版(Response Evaluation Criteriation in Solid Tumours, RECIST v1.1)评价客观疗效，按照不良反应通用术语标准4.0版(Common Terminology Criteria for Adverse Events v4.0, CTC AE v4.0)评估用药期间发生的不良事件。

**结果:**

该患者多线治疗后，颅内病灶控制欠佳，脑脊液中发现异型细胞，EGFR基因19外显子缺失突变，服用厄洛替尼4周后，颅内客观疗效为部分缓解(partial response, PR)，颅外客观疗效为疾病稳定(stable disease, SD)，颅内无进展生存期(progression-free survival, PFS) 10.5个月，总生存期(overall survival, OS) 11个月。主要不良反应为皮疹(1级)。

**结论:**

脑脊液检测EGFR突变可为制定治疗策略提供理论支持，根据不同的突变状态给予小分子靶向药物联合化疗，可分别控制颅内及颅外病灶。

## Abstract (en)

**Background and objective:**

One of the most ofen distance metastasis site of non-small cell lung cancer (NSCLC) is brain and the standard treatment of brain metastasis was radiotheraphy including whole brain irradiation (WBI) and stereotactic radiotherapy (SRT). It has been reported that epidermal growth factor receptor tyrosine kinase inhibitors (EGFR-TKIs) had the active response in brain metastasis of lung cancer. In the present study, we reported one case of EGFR 19el in cerebrospinal fluid tested by ARMS got partial response given erlotinib.

**Methods:**

Cerebrospinal fluid was collected through lumbar puncture, then cast-off cells and *EGFR* mutation was analysed. Erlotinib was given with dose of 150 mg, *qd*. Objective response was evaluated by Response Evaluation Criteriation in Solid Tumours (RECIST) v1.1 and adeverse events were evaluated according to Common Terminology Criteria for Adverse Events v4.0 (CTC AE v4.0).

**Results:**

Heterocyst cells were found in cerebrospinal fluid and *EGFR* mutation was tested as 19del. Te patient achieved partial response (PR) of brain metastasis and the effective response in lung was stable disease (SD) afer 4 weeks of erlotinib. Te progression-free survival (PFS) and overall survival (OS) of brain metastasis was 10.5 months and 11 months respectively. Te main adverse event was rash (Grade Ⅰ).

**Conclusion:**

It was feasible to test *EGFR* mutation in cerebrospinal fluid and the combination of erlotinib with chemotheraphy would be an appropriate choice to those lung cancer patients who had brain metastasis harboring *EGFR* sensitive mutation.

肺癌是我国发病率和死亡率最高的恶性肿瘤之一，其中非小细胞肺癌(non-small cell lung cancer, NSCLC)约占85%^[1]^。研究^[2]^证实，表皮生长因子受体(epidermal growth factor receptor, *EGFR*)突变是NSCLC的重要驱动基因之一，NSCLC中*EGFR*敏感突变的比例约为50%。近十年来，小分子靶向药物EGFR酪氨酸激酶抑制剂(tyrosine kinase inhibitors, TKIs)在临床的广泛应用使晚期NSCLC的总生存期(overall survival, OS)从不到1年延长至超过30个月，可谓"里程碑式的进展" ^[3-5]^。临床实践中，EGFR-TKIs的治疗已经涵盖了NSCLC治疗的一线、维持、二线及以后的治疗。

脑是肺癌常见的转移部位，出现脑转移后，标准治疗仍为放疗，包括全脑照射(whole brain irradiation, WBI)和立体定向照射(stereotactic radiotherapy, SRT)。有研究显示EGFR-TKI可透过血脑屏障，发挥抗肿瘤作用，但收集脑脊液进行*EGFR*突变检测的报道并不多。本文报道一例肺腺癌患者，在首次TKI及多程化疗失败后，检测脑脊液*EGFR* 19del，给予二次TKI联合化疗治疗有效，为TKIs治疗肺癌脑转移提供了确切的理论依据及治疗思路。

## 1 临床资料

患者，王某某，女性，43岁，河北邢台人，农民，从不吸烟，无家族肿瘤患病史。2011年3月因"咳嗽、胸闷2个月，加重1周"入我院。查体：美国东部肿瘤协作组体力状况(Eastern Cooperative Oncology Group performance status, ECOG PS) 1，左锁骨上可扪及2 cm左右肿大淋巴结，质韧，无明显压痛。左肺下叶呼吸音低，余肺呼吸音清，未闻及明显干湿性罗音。胸部增强计算机断层扫描(computed tomography, CT)示"左肺占位，纵隔及左肺门淋巴结肿大伴阻塞性肺不张，左侧胸腔积液"，浅表淋巴结B超示"右颈下段及双锁骨上多发肿大淋巴结，大者1.3 cm×0.9 cm"，骨扫描示"胸1椎体异常浓聚，考虑转移"，腹部增强CT及脑磁共振成像(magnetic resonance imaging, MRI)未见占位性病变。行支气管镜检查，刷片可见"异型细胞"，3月24日行左锁骨上淋巴结穿刺活检，病理回报"腺癌"，因此明确诊断为：左肺腺癌(T2N3M1b，Ⅳ期)。

治疗经过(表 1)：入组LUX-Lung6临床研究，检测*EGFR*基因外显子19缺失突变，随机分入试验组，于2 011年5月6日开始一线治疗：口服BIBW2992(afatinib，阿法替尼)，40 mg，每日一次。服药6周时复查CT，按照实体瘤疗效评价标准(Response Evaluation Criteria in Solid Tumors，R ECIST) v1.0评价客观疗效为部分缓解(partial response, PR)(图 1A，图 1B)，此后每6周复查，维持疗效PR，2012年4月1日复查胸部CT示病情进展，停止afatinib治疗。于2012年4月-2012年8月进行二线化疗：培美曲塞二钠(Pem, 500 mg/m^2^, d1) +顺铂(DDP，75mg/m^2^，分2天)，共6周期，最佳疗效PR(图 1C，图 1D)，PFS为12个月。2013年3月常规复查提示肺内病灶进展，且出现无症状脑转移，转移部位位于左侧小脑半球。2013年3月至6月给予三线化疗：多西他赛(DOC, 75 mg/m^2^, d1) +卡铂(CBP, AUC=5, d1)，4周期后给予多西他赛单药化疗2周期，肺内(图 1E，图 1F)及颅内最佳疗效均为PR(图 2A，图 2B)，PFS为7个月。2013年11月复查提示肺内及颅内病灶增大，脑转移无症状，遂给予四线治疗：培美曲塞二钠(Pem, 500 mg/m^2^, d1) +卡铂(CBP, AUC=5, d1)，2周期后评价疗效：肺部病情稳定(stable disease, SD)，颅内病灶PR(图 3A，图 3B)，但患者出现头痛，伴呕吐，不排除脑膜转移可能。于2013年12月29日行腰穿，脑脊液清亮，压力300 mmH_2_O，涂片可见异型细胞，突变扩增阻滞系统(amplification refractory mutation system, ARMS)(厦门艾德ADx-ARMS)检测脑脊液*EGFR*突变示"19外显子缺失突变"，同法外周血*EGFR*突变检测为阴性。因此于2013年12月30日开始给予四线第3周期PCb化疗，同时联合厄洛替尼(erlotinib, tarceva) 150 mg/d治疗。服药第3天时头痛、呕吐等颅高压症状缓解。2014年1月30日(厄洛替尼治疗1个月)复查示肺部病灶SD，颅内病灶PR(图 3C)，服药2个月后复查CT示肺部病灶病情进展(progressive disease, PD)，颅内转移灶疗效维持PR。自2014年3月28日起给予五线多西他赛(75 mg/m^2^, d1)单药化疗4周期联合厄洛替尼150 mg/d治疗，最佳疗效SD(肺内病灶及颅内病灶均维持稳定，无新发病灶)，PFS为7个月，厄洛替尼单药PFS为10个月。2014年10月9日患者出现头痛、视物模糊、呕吐，ECOG PS 3分，入院后查胸部CT示"左肺病灶较前增大"(图 4A)，脑MRI示"左侧小脑半球占位较前增大，脑膜部分强化"(图 4B)，给予脱水、激素等缓解症状，取得患者知情同意后再次进行腰穿，脑脊液清亮，压力超过330 mmH_2_O，涂片查见异型细胞，检测*EGFR*突变示"19del & T790M突变"，提示耐药。于2014年10月12日开始全脑放疗，计划剂量Dt 4, 000 cGy/20 f/4 w，放疗4次后，患者颅高压症状略缓解，但放弃后续治疗返回当地，于2014年10月30日死亡。总生存期3年7个月。

**1:** 患者的治疗、反应及无进展生存期 Treatment, response and PFS of the patient

	Time	Therapeutic programs	ORR	PFS(mo)
1^st^ line	2011.4-2012.32	Afatinib	PR	11
2^nd^ line	2012.4-2012.8	Pem+Cis	PR	12
3^rd^ line	2013.3-2013.6	DOC+CBP	PR	7
4^th^ line	2013.12-2014.3	Pem+CBP, 3 cycles	Lung SD	PFS 4 months
		Then conbined with Tarceva	Brain PR	
5^th^ line	2014.3-2014.6	DOC+Tarceva	Lung SD	PFS in Lung: 3 months
			Brain PR	PFS in Brain: 10 months
Local treatment	2014.10.12	Whole brain irradiation	Uncompleted	-
		Dt 40 Gy/20 f/4 wk		
SD: stable disease; PFS: progression-free survival.				

**1:**
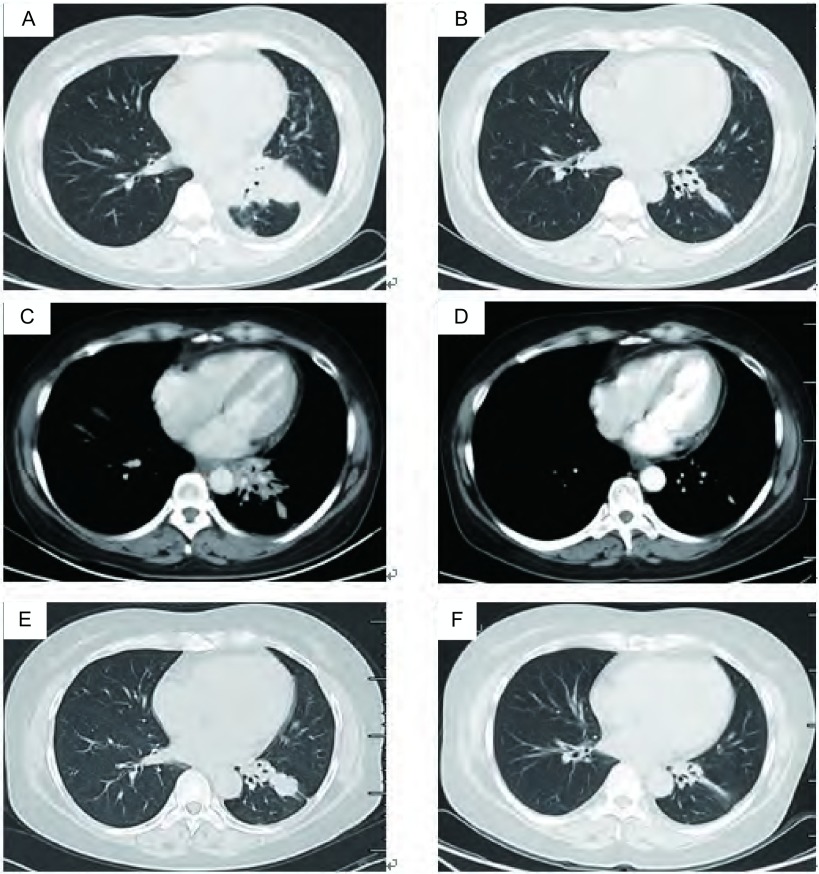
阿法替尼及化疗前后肺部靶病灶的变化。A：阿法替尼治疗前；B：阿法替尼治疗6周后，客观疗效PR；C：二线培美曲塞+顺铂化疗前；D：二线培美曲塞+顺铂化疗2周期后，客观疗效PR；E：三线多西他赛+卡铂化疗前；F：三线多西他赛+卡铂化疗2周期后，客观疗效PR。 Patient's objective response ratio (ORR) of afatinib and chemotheraphy. A: Before given afatinib; B: After six weeks of afatinib and the ORR was partial response (PR); C: Before treatment of second line of pemetrexed and cisplatin; D: After six weeks of second line chemotheraphy and the ORR was PR; E: Before treatment of third line of docetaxel and carboplatin; F: After six weeks of third line chemotheraphy and the ORR was PR.

**2:**
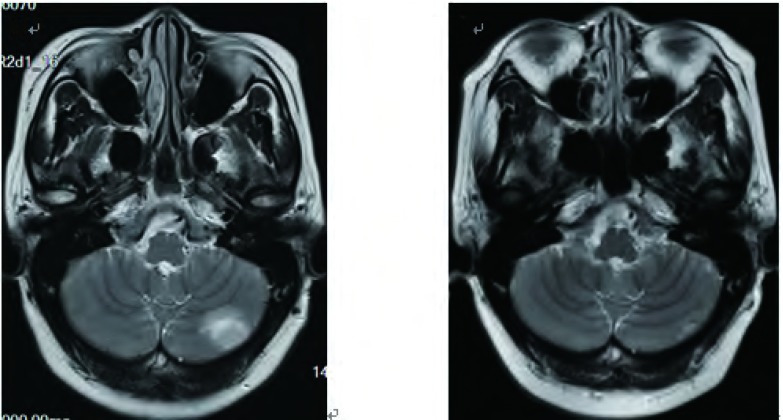
化疗对脑转移病灶的疗效。A：三线多西他赛+卡铂化疗前；B：三线多西他赛+卡铂化疗2周期后，左小脑病灶明显缩小，客观疗效PR。 The response of chemotheraphy to brain metastasis. A: Before treatment of third line of docetaxel and carboplatin; B: After 2 cycles of docetaxel and carboplatin, the left cerebellum metastasis was shrinked obviously and objective response was PR.

**3:**
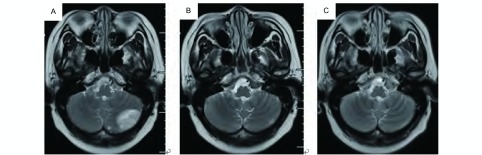
四线化疗及厄洛替尼对脑转移病灶的疗效。A：四线培美曲塞+卡铂化疗前；B：四线培美曲塞+卡铂化疗2周期后，颅内疗效PR；C：厄洛替尼治疗1个月后，颅内病灶进一步缩小，且颅高压症状消失。 The response of fourth line chemotheraphy and tarceva in brain metastasis. A: Before fourth line treatment of pemetrexed and carboplatin; B: After 2 cycles of chemotheraphy, the objective response was PR; C: After one month of tarceva, the brain metastasis was shrinked smaller than before with symptom of intracranial hypertension disappeared.

**4:**
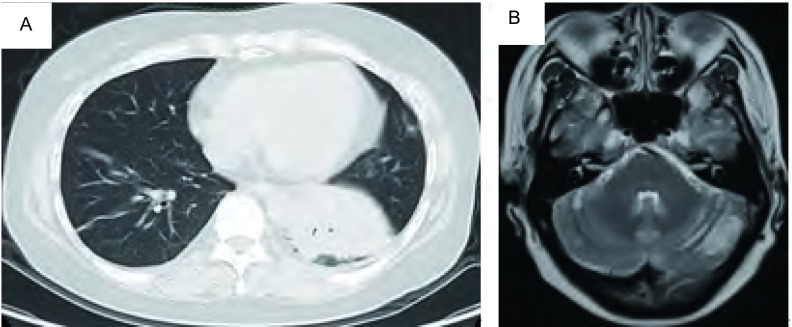
2014年10月病情进展后。A：左肺病灶较前增大；B：左侧小脑半球病灶较前明显增大。 Progression of disease in October, 2014. A: The tumor in left lung was larger than before; B: The metastasis of left cerebellum was larger obviously.

## 2 讨论

自2008年IPASS研究发表以来，先后有八项大型多中心、随机、对照临床研究证实：对于*EGFR*敏感突变的肺癌患者，一线给予EGFR-TKI治疗，无论客观缓解率(objective response rate, ORR)还是PFS，均较化疗有显著优势，因此早在2010年，美国国立综合癌症网络(National Comprehensive Cancer Network, NCCN)推荐对腺癌患者常规进行EGFR检测，阳性患者建议优先TKI治疗。2013年发表的LUX-Lung3^[5]^临床研究显示，作为二代不可逆的TKI，afatinib(阿法替尼)治疗EGFR敏感突变的肺癌，与化疗相比，ORR、PFS及OS均有显著优势。该患者为EGFR 19外显子缺失，恰逢LUX-Lung6在中国招募受试者，其接受了afatinib的治疗，客观疗效PR，一线PFS 11个月，提示明显获益。

TKI耐药后的治疗取决于患者一般状况、治疗时机及可检测到的耐药机制。对于一线阿法替尼失败的患者，在PS 0分-1分时，标准化疗为首选治疗方案，且对于*EGFR*突变的肺癌，化疗的ORR较*EGFR*野生型患者升高。该患者在afatinib治疗失败后的27个月内先后接受了三种方案共19个周期的化疗，且前两个化疗方案的客观疗效均为PR，这提示恰当的化疗是较长OS的重要贡献力量。

理想情况下，EGFR-TKI耐药后，需再次活检，明确耐药机制，据此制定后续治疗方案，但在实际临床实践中，受限于患者依丛性及可穿刺部位的不确定性，并非每个EGFR-TKIs耐药的患者均能接受再次活检，尤其是脑脊液EGFR突变，因取材难度及风险增加，难以开展常规检测^[6, 7]^。而随着*EGFR*突变患者OS的延长，脑转移的几率随之增加，如何提高脑转移病灶的局部控制率成为肺癌患者全程管理中需要解决的难题。本文报道的患者在四线化疗过程中，尽管影像学评价颅内转移病灶客观疗效为PR，但出现颅高压症状，遂进行腰穿，发现脑脊液中有异型细胞，更有提示意义的是，脑脊液检测EGFR为19del，而同样方法检测外周血*EGFR*为野生型，尽管彼时没有肿瘤组织的检测结果，且ARMS方法检测外周血*EGFR*突变的敏感性仍有待提高，但此次检测脑脊液和外周血*EGFR*突变状态的不一致初步提示肿瘤异质性的存在。2015年6月，对保存的该患者的脑脊液和外周血ctDNA用数字PCR(ddPCR)方法进行了回顾性复测，结果同前，仍为脑脊液为*EGFR*突变型，外周血为野生型。随着检测方法的优化及检测成本的降低，未来ddPCR和二代测序会越来越多地用于临床，检测结果会更精准地指导临床治疗，对特殊部位体液(如脑脊液)的检测也会成为常规。

理论上认为小分子TKI可以透过血脑屏障，且已有较多报道证实了TKIs治疗肺癌脑转移的可行性^[8-10]^。因此，在化疗基础上，联合应用厄洛替尼，以期控制颅内病灶。服药后，患者首先表现出颅高压症状的减轻至消失，1个月后复查提示颅内疗效PR，2个月后颅内疗效维持PR，而肺内病灶增大，这提示先前脑脊液与外周血分别检测*EGFR*突变的状态不一致，与颅内及肺内病灶不同的疗效相符合。该患者出现颅高压症状后检测脑脊液EGFR示19外显子缺失突变，服用靶向药物10个月后颅内病灶较前增大，再次查脑脊液*EGFR*突变示19del与T790M并存，从机制上明确了耐药原因，令人遗憾的是，当时国内三代EGFR-TKI的抑制剂(如AZD9291、CO-1686、avitinib等)尚未开展临床研究，倘若目前有类似的患者，定能从更精准的治疗中获益。

总之，该例患者初诊时为肺腺癌Ⅳ期，先后从TKI及化疗中获益，并且通过分子诊断的方法明确了驱动基因的存在，根据基因突变的不同给予不同的治疗，使不同部位的肿瘤均得到控制，总生存期达3年7个月，这提示我们，病理联合分子诊断指导下的肺癌治疗的全程管理是患者OS延长的保证。
